# Latitudinal variation in soil biota: testing the biotic interaction hypothesis with an invasive plant and a native congener

**DOI:** 10.1038/s41396-018-0219-5

**Published:** 2018-07-16

**Authors:** Xinmin Lu, Minyan He, Jianqing Ding, Evan Siemann

**Affiliations:** 1 Key Laboratory of Aquatic Botany and Watershed Ecology, Wuhan Botanical Garden, Chinese Academy of Sciences, Wuhan, Hubei China; 2 School of Life Sciences, Central China Normal University, Wuhan, Hubei China; 3 College of Life Sciences, Henan University, Kaifeng, Henan China; 4 Biosciences Department, Rice University, Houston, TX USA

## Abstract

Soil biota community structure can change with latitude, but the effects of changes on native plants, invasive plants, and their herbivores remain unclear. Here, we examined latitudinal variation in the soil biota community associated with the invasive plant *Alternanthera philoxeroides* and its native congener *A. sessilis*, and the effects of soil biota community variation on these plants and the beetle *Agasicles hygrophila*. We characterized the soil bacterial and fungal communities and root-knot nematodes of plant rhizospheres collected from 22 °N to 36.6 °N in China. Soil biota community structure changed with latitude as a function of climate and soil properties. Root-knot nematode abundance and potential soil fungal pathogen diversity (classified with FUNGuild) decreased with latitude, apparently due to higher soil pH and lower temperatures. A greenhouse experiment and lab bioassay showed native plant mass, seed production, and mass of beetles fed native foliage increased with soil collection latitude. However, there were no latitudinal patterns for the invasive plant. These results suggest that invasive and native plants and, consequently, their herbivores have different responses to latitudinal changes in soil-borne enemies, potentially creating spatial variation in enemy release or biotic resistance. This highlights the importance of linking above- and below-ground multitrophic interactions to explore the role of soil biota in non-native plant invasions with a biogeographic approach.

## Introduction

Biotic interactions can influence species distribution, community structure, and non-native species invasions [1, 2]. Understanding how biotic interactions respond to environmental changes is critical for the current and future conservation of biodiversity [3]. Biogeography is central to synthesizing large-scale patterns of species interactions and their responses to environmental changes, and provides a window to assess the impacts of changing biotic interactions on species distributions [4]. The Latitudinal Biotic Interaction Hypothesis posits that biotic interactions are more intense at low than at high latitudes because a more benign climate and higher species diversity at low latitudes lead to stronger interactions [5, 6]. This theory has been tested extensively on antagonistic and mutualistic interactions between native species, with many studies showing higher levels of herbivory, predation, or parasitism at low than at high latitudes [5, 7]; however, other studies have found no such pattern [8, 9]. Most of these studies have emphasized above-ground interactions, largely overlooking plant–soil biota interactions and the indirect, plant-mediated impacts of soil biota on above-ground herbivores [5, 8, 9].

Most non-native invasive plants are distributed across a wide range of latitudes in their invaded ranges, and thus might experience varying biotic interactions, especially during range expansion [10, 11]. Biotic factors, such as native pathogens and herbivores or native competitors, play an important role in non-native plant invasions [2]. Several theories incorporating biotic interactions, such as the Enemy Release Hypothesis [12] and the Biotic Resistance Hypothesis [13], have been developed to explain plant invasions. Integrating these theories with the Latitudinal Biotic Interaction Hypothesis can provide a foundation for understanding the spatial heterogeneity of plant invasions and the underlying causes. If latitudinal variation in the biotic interactions of native plants differs from that of invasive plants, then large-scale heterogeneity in the strength of enemy release or biotic resistance could affect plant invasions [14]. Latitudinal variation in invasive plant-above-ground herbivore interactions and the possible causes (such as local adaptation, phenotypic plasticity or apparent competition) have recently received attention [15–19]. For instance, in North America, herbivores on the native genotype *Phragmites australis* decrease with latitude, but no latitudinal pattern exists for the invasive genotype, potentially resulting in decreasing enemy release intensity and increasing biotic resistance intensity with latitude for the invasive genotype [18, 19]. More generally, differences in selection pressures for native and invasive plants and the length of time that each group is present at a location may cause evolutionary differences that lead to non-parallel gradients with increasingly intense plant–soil biota interactions at lower latitudes for native plants but not invasive plants. It remains unclear how plant–soil biota interactions and their indirect effects on above-ground plant–herbivore interactions change with latitude for closely related native and invasive plants.

Soil biota can have profound effects on non-native plant invasions [20]. Studies at the local-scale (e.g., a single community or ecosystem) have found that invasive plants often are less negatively affected than co-occurring native plants by soil microbes and herbivores, indicating the former escapes soil-borne enemies [21]. This may indicate that invasive plants possess more effective defenses against generalist enemies [22] or novel antimicrobial chemicals (the Novel Weapon Hypothesis) [23]. In addition, some invasive plants benefit from high levels of colonization by local mutualists [24]. Soil biotic effects on plants can be negative or positive, depending on the community composition of the soil biota, especially the relative abundances of mutualists (e.g., arbuscular mycorrhizal fungi, AMF), herbivores (e.g., root nematodes) and pathogens [25] that vary with latitude in response to changes in soil properties and climate [26, 27]. For example, the diversity of soil fungi and fungal pathogens increases towards the equator [26]. In parallel, studies with native plants have found greater negative impacts of soil biota at low than at high latitudes [28, 29]. In contrast with latitudinal patterns of biotic interactions for native plants that reflect the co-adaptation of interacting species over evolutionary time, invasive plant–soil biota interactions are characterized by evolutionary novelty [16]. Therefore, the latitudinal variation in plant–soil interactions that native plants experience [28, 29] is expected to be absent for invasive plants, potentially creating large-scale heterogeneity in the strength of enemy release or biotic resistance.

Increasing evidence has indicated that the effects of soil biota on plants could cascade to above-ground herbivores [30, 31]. Empirical studies have indicated that soil biota can alter a host plant’s palatability or defenses, which may change host choice behavior and the performance of above-ground herbivores, potentially modifying plant-above-ground herbivore interactions and, in turn, plant invasions [31, 32]. Thus, latitudinal variation in soil biota community composition may also indirectly determine the performance of above-ground herbivores by affecting invasive and native plant hosts. However, existing studies on changes in plant–soil biota interactions with latitude have employed a strictly bi-trophic framework [28, 29], potentially overlooking indirect effects of the soil biota on above-ground herbivores.

Here, we characterized bacterial and fungal communities and root-knot nematodes in soils collected along a latitudinal gradient (from 22 °N to 36.6 °N) in continental China. Then, we experimentally estimated the direct impacts of the soil biota on the invasive plant *Alternanthera philoxeroides* (alligator weed) and its native congener *A. sessilis*, as well as the indirect impacts of the soil biota on the biocontrol beetle *Agasicles hygrophila* (alligator weed flea beetle) mediated by these plant species. This system is well suited for this kind of study. First, *A. philoxeroides* co-occurs with *A. sessilis* along its entire latitudinal range and their competition changes with latitude [33, 34]. Second, *A. hygrophila* and the native root-knot nematode *Meloidogyne incognita* are the main above- and below-ground herbivores attacking these plant species [33]. Third, interactions with herbivores differ for these plant species, as the invasive plant has more effective defenses against both above- and below-ground herbivores [35]. Here, we characterized soil bacterial and fungal community compositions by high-throughput sequencing, used climate and soil physical properties to investigate the causes of soil biota community variation, and explored links between soil biota community composition and performance of the plant and beetle species in a greenhouse experiment and lab bioassay. Specifically, we asked: (1) Does the soil biota community (bacteria, fungi, root-knot nematodes) associated with the invasive and native plants change with latitude? (2) If so, which climate or soil properties influence latitudinal variation in soil biota communities? (3) How do soil biotic effects on the invasive vs. native plant and beetle change with soil collection latitude? We predicted that soil biota community diversity and effects of the soil biota on native plants and beetles consuming their foliage would decrease with increasing latitude (consistent with the Latitudinal Biotic Interaction Hypothesis). However, the effects of soil biota on the invasive plant, which lacks a long history of association with these soil communities, would not vary with latitude.

## Materials and methods

### Study species

*Alternanthera philoxeroides* (Amaranthaceae), which is native to South America, is a global invader with an expanding range [36, 37]. In continental China, it propagates only via buds and stems, is distributed from 21 °N to 36.8 °N [37], and has low genetic diversity [38]. *Alternanthera sessilis*, native to China, can propagate via seeds or stem buds [34]. The latitudinal range of *A. sessilis* fully overlaps with that of *A. philoxeroides* in continental China [33]. Both species can form dense mats on land. *Alternanthera sessilis* mainly has fine roots with thin cuticles and high surface area per biomass, while *A. philoxeroides* mainly has coarse roots with thick cuticles and low surface area per biomass [35]. Therefore, *A. sessilis* is more susceptible than *A. philoxeroides* to root infection [35].

*Agasicles hygrophila* (Coleoptera: Chrysomelidae) is a South American herbivore with a narrow host range (*Alternanthera*) that was first released in China in 1986 to control *A. philoxeroides* but also attacks the non-target plant *A. sessilis* [33]. The beetle’s range has expanded to 30.8 °N in China in the past few decades, possibly in part due to increases in temperature [37]. *Meloidogyne incognita* (Tylenchida: Heteroderidae), a generalist root-knot nematode, is the most widespread and common nematode pest of agricultural and semi-natural systems in tropical and subtropical regions (www.cabi.org/isc/datasheet/33245).

### Soil collection

In May 2015, we collected rhizosphere soils of *A. philoxeroides* and *A. sessilis* monocultures at 16 sites from 22 °N to 36.6 °N, covering the whole latitudinal range of *A. philoxeroides* in continental China (Supplementary Fig. S1). Along the gradient, annual minimum temperature (−1.17 °C per degree, *R*^2 = ^0.9591, *P* *<* 0.0001, linear regression) and annual precipitation (−115.80 mm per degree, *R*^2 = ^0.9432, *P* *<* 0.0001) decreased with latitude, but there was no latitudinal pattern for annual maximum temperature (*R*^*2* *=* ^0.2009, *P* *=* 0.0817; data from the National Meteorological Center of China; Supplementary Fig. S1A, B). To account for spatial heterogeneity, we collected soils at five locations (10 km apart) for each of the 16 sites and 15 soil cores (2.5 cm in diameter, 10 cm in depth) for each plant species at each location. We immediately placed the soil cores in plastic bags and kept them at 4 °C. We finished soil collection within 2 weeks; thus, soil samples were stored for different periods up to 14 days, but this likely had a negligible impact based on the reports of other studies [28, 29]. In a lab, we sieved (2.5 mm) and mixed all 75 soil samples from each site and plant species to homogenize soil and remove plant tissues. This soil mixing approach might overlook soil biota community variation at small spatial scales and can generate falsely precise statistical estimates [39]. Then, we divided the soil sample from each site and rhizosphere species combination into four parts that we: (1) used to measure pH values, (2) air dried for chemical analysis, (3) stored at −20 °C for DNA extraction and sequencing, or (4) used for the greenhouse experiment. We cleaned and sterilized all equipment used in these lab procedures and experiments by bleach immersion (24 h) between soil types (i.e., between each site and rhizosphere species combination).

We measured soil pH with a pH meter (PHSJ-3F, Shanghai Jingke Company). We analyzed soil total nitrogen (TN) and carbon (TC) contents (C/N analyzer, Vario MAX CN, Elementar Analysensysteme GmbH, Germany), total phosphorus (TP) (inductively coupled plasma atomic emission Spectrometry, ICP-AES), available (AP) phosphorus (Spectrophotometric molybdenum blue method), available nitrogen (AN) (Alkali N- proliferation method), and organic carbon (OC) (Tyurin titrimetric method).

### DNA extraction, PCR, and sequencing

Soil DNA was extracted with the Ezup genomic DNA extraction kit (Sangon Biotech, China). Bacterial *16S rRNA* genes were amplified with the universal primers 515F and 909R, and fungal *ITSF1* genes were amplified with the forward primer ITSF2_KYO2 and reverse primer ITSF3. Two parallel 25 μl PCR reactions were conducted for each DNA sample, and the PCR products were purified and pooled together for each DNA sample. Purified libraries were diluted, denatured, re-diluted, mixed with PhiX (equal to 30% of the final DNA amount) and sequenced with an Illumina MiSeq system [see details in ref. 40].

All sequences were analyzed with QIIME Pipeline—Version 1.7.0, and sorted to each sample based on their unique barcodes. High-quality sequences were used for downstream analysis, and chimera sequences were removed with the UCHIME algorithm [41]. All sequences were clustered into operational taxonomic units (OTUs) with a 97% identity cutoff. Bacterial 16s rRNA OTUs taxonomy was assigned with the Ribosomal Database Project classifier [42]. Fungal ITS sequences were blasted against the GenBank nucleotide database. DNA sequencing data are available at the European Nucleotide Archive (ENA) with the accession numbers PRJEB22110 and PRJEB22111. We classified whether the fungal OTUs were likely to be potential plant pathogens with FUNGuild at the “possible” confidence level [43]. FUNGuild could only assign a fungus as a potential pathogen for some plants [43]. However, this coarse categorization was necessary because there was little information on soil pathogens for the two studied plant species. All Glomeromycota were classified as AMF.

### Greenhouse experiment

To test the direct impacts of soil bacteria, fungi, and nematodes on *A. philoxeroides* vs. *A. sessilis*, we conducted a greenhouse experiment in Wuhan from June to December 2015. We collected 0–10 cm top-soil from a local field and sterilized it with gamma rays. Then, for 15 of the sites across latitudes, we mixed the rhizosphere soil samples of either the native or invasive plants with sterilized top-soil at the ratio of 1:4 (v/v) to reduce potential impacts of soil abiotic properties on plant performance. One site did not have sufficient soil after screening (Table S1). We put the soil mixtures into 15 L bleach-sterilized pots.

One week later, we transplanted individually into pots similar-sized *A. philoxeroides* and *A. sessilis* plants that we had cultivated by putting cut stems into sterilized soil in a greenhouse. We only used *A. philoxeroides* and *A. sessilis* collected from local populations (near Wuhan Botanical Garden, middle latitude, 30.5 °N) to minimize potential impacts of plant genetic and/or maternally inherited variation on experimental results. The experiment had 240 pots: 15 soil collection sites × 2 soil types (rhizosphere of *A. philoxeroides* vs. rhizosphere of *A. sessilis*) × 2 plant species (*A. philoxeroides* vs. *A. sessilis*) × 4 replicates. Throughout the experiment, we watered plants weekly with filtered water (particles with diameter > 0.1 µm were removed).

At the end of the experiment, we counted the flowers and the seeds in ten randomly selected flowers on each *A. sessilis* plant to estimate total seed number for each individual. We also collected 20 fully-opened, similar-sized leaves from the top of each plant (each plant had hundreds of leaves). We mixed leaves from the same treatment and stored them at 4 °C for the beetle bioassay. Then, we harvested plants, rinsed roots to remove soil, and counted *M. incognita* root knots. We dried plants at 80 °C for 48 h and weighed them.

### Lab bioassay

To test the indirect impacts of soil bacteria, fungi, and nematodes on beetles via the native vs. invasive plants, we conducted a lab bioassay. Lab bioassays that feed leaves to insects under controlled temperature and photoperiod conditions are a powerful way to test how variations in plant palatability or defense impact insect performance [44]. We collected *A. hygrophila* beetles from a local field, reared them on *A. philoxeroides* in mesh cages for one generation, and used their offspring for bioassays. We transferred newly hatched larvae into petri dishes (9 cm diameter) lined with moist filter paper and placed a single intact leaf into each dish. The leaves of 5 of the 60 treatment combinations were destroyed by a refrigerator malfunction (Supplementary Table S1), leaving 55 treatment combinations that were replicated ten times. We maintained dishes at 28 °C with a natural light/dark photoperiod (14:10) and kept the filter papers moist. Every other day, we replaced each leaf with an intact leaf from a plant from the same treatment. One week later, we weighed the fresh mass of surviving beetles after 24 h of starvation.

### Data analysis

We used ANCOVAs (i.e., ANOCOVAs) to examine the effects of soil type (categorical variable: rhizosphere of *A. sessilis* vs. rhizosphere of *A. philoxeroides*), latitude (continuous variable), and their interaction on soil chemistry variables. We used Cohen’s *f*^2^ to estimate local effect sizes. We considered effects to be significant when the *P*-values were <0.05 and classified the effect magnitudes as small (0.10 < *f*^2^ ≤ 0.25), medium (0.25 < *f*^2^ ≤ 0.40), or large (0.40 < *f*^2^) following previously published guidelines [45]. We used principal component analysis (three axes) to examine variation in environmental variables (i.e., climate and soil properties).

For soil bacterial and fungal communities, we used principal component analysis (PCA) to examine taxonomic composition variation within groups (all fungal OTUs, AMF OTUs, potential fungal pathogen OTUs, bacteria OTUs; three axes in every case). We chose PCA to represent community variation with a small number of summary variables that vary linearly with latitude. We conducted ANCOVAs to determine which site characteristics predicted variation in PCA axes for each group. Starting models included continuous soil chemistry (TC, TN, TP, C:N, C:P, N:P, AN, AP, OC, pH) and climate variables (Tmin, Tmax, precipitation) and the categorical variable soil type. Stepwise, forward, and backward models selected the same final model for each PCA axis.

To examine the dependence of soil taxonomic composition on latitude and soil type, we performed ANCOVAs using pairwise dissimilarity indices (two separate response variables), differences in latitude (continuous predictor), and combinations of soil types (categorical predictor). For every pair of data points *p* and *q*, we calculated their Euclidean distance *d* (*p*, *q*), which reflects both OTU presence/absence and changes in OTU dominance as sqrt([*p*_1_ − *q*_1_]^2^ + [*p*_2_ − *q*_2_]^2^ + … + [*p*_*n*_ − *q*_*n*_]^2^), where _*pi*_ is the proportion of reads that were OTU_*i*_ for sample *p*, *q*_*i*_ is the proportion of reads that were OTU_*i*_ for sample *q*, and *n* is the total number of OTUs across all samples. We calculated their Jaccard’s dissimilarity index, which uses OTU presence/absence only and, therefore, only includes changes in species composition. Jaccard’s dissimilarity index is calculated as one minus the number of OTUs that occurred in both *p* and *q* divided by the total number of OTUs that occurred in *p* and/or *q*. We calculated the difference between their latitudes of collection and assigned them a category to indicate whether a pair of samples were both from *A. philoxeroides* soils, both from *A. sessilis* soils, or from different soil types.

We used ANCOVAs to examine the effects of soil collection latitude, plant species (categorical variable), soil type, and their interactions on plant mass. We conducted similar analyses that examined effects on number of root knots and beetle mass. We fit models for numbers of seeds produced by *A. sessilis*. We performed an ANCOVA (response variable was the number of surviving beetles out of ten initial larvae with a binomial error distribution and a logit link function) to test whether beetle survival odds varied with the same set of predictors used in the beetle mass analysis.

To explore mechanisms for latitudinal effects on plant and beetle performance, we conducted a path analysis. First, we examined covariance among environmental variables. Because several temperature and precipitation variables were strongly correlated (*r* > 0.95), we only included Tmin and Tmax, which were independent of other climate variables. Second, we used regression with standardized coefficients to quantify the effect of latitude on Tmin and Tmax plus soil pH. Third, we conducted additional regressions to examine the effects of these climate and soil variables on microbial variables (numbers of total fungal, AMF, fungal pathogen, and bacterial OTUs) and below-ground herbivory (number of nematode root knots). Fourth, we performed a regression to test the dependence of *A. sessilis* seed production on soil microbial variables, below-ground herbivory, and soil abiotic variables. Because *A. philoxeroides* only reproduces vegetatively in China, we used plant mass to examine its responses to these variables. Finally, we examined the dependence of beetles on plant variables. In every regression, we only included significant paths. We only show results for variables that varied with latitude or impacted plant performance.

All analyses were conducted with SAS 9.4 (SAS Institute, Cary, North Carolina, USA).

## Results

Soil pH increased by 16.5% along the latitudinal gradient (*F*_1, 29_ = 12.96, *P* = 0.0012, *f*^2^ = 0.463) but the rate of increase did not differ between soil types (rhizosphere of *A. sessilis* vs. rhizosphere of *A. philoxeroides*) (Supplementary Table S2 and Supplementary Fig. S2). There was no latitudinal pattern for TC, TN, TP, C:N, C:P, N:P, AN, AP, or OC (all *P* *>* 0.05, Supplementary Table S2 and Supplementary Fig. S3).

**Fig. 1:**
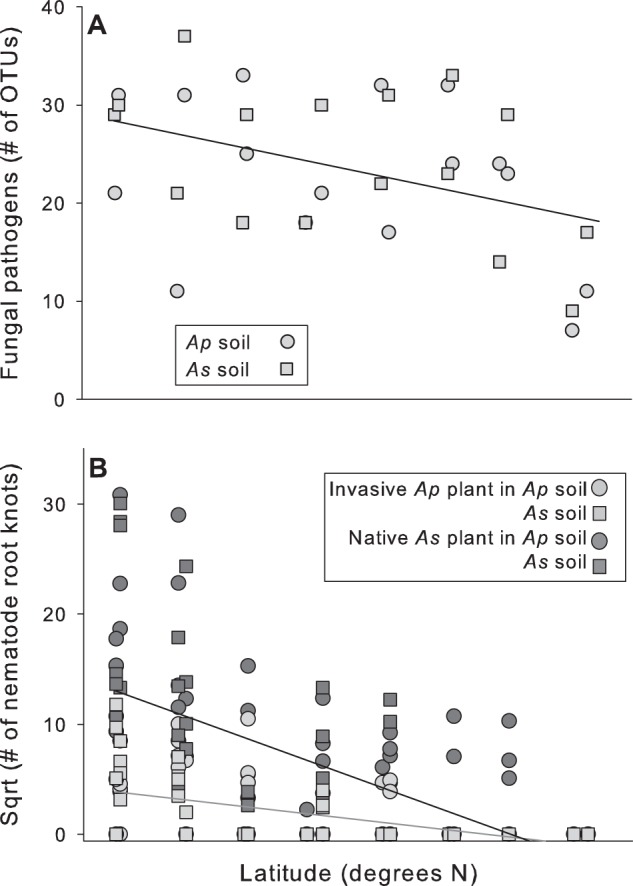
**a** The number of OTUs classified as potential fungal pathogens from invasive *A. philoxeroides* (circles) and native *A. sessilis* (squares) rhizosphere soils collected along a latitudinal gradient. Lines indicate the effects of latitude. **b** The effects of latitude on the number of nematode knots on roots of invasive *A. philoxeroides* (light gray) and native *A. sessilis* (dark gray) plants grown in these soils

The number of potential fungal pathogen OTUs decreased by 35.2% (*F*_1, 29_ = 7.33, *P* *=* 0.0114, *f*^2^ = 0.262, Fig. 1a) and the number of bacterial OTUs increased by 14.6% (*F*_1, 29_ = 6.55, *P* *=* 0.0162, *f*^2^ = 0.234) along the latitudinal gradient, but the numbers of AMF (*F*_1, 29_ = 0.02, *P* *=* 0.9028) and total fungal (*F*_1, 29_ = 0.76, *P* *=* 0.3912) OTUs did not change with latitude (Supplementary Table S2). Soil type did not affect any of these variables or change the effect that latitude had on them (Supplementary Table S2).

**Fig. 2:**
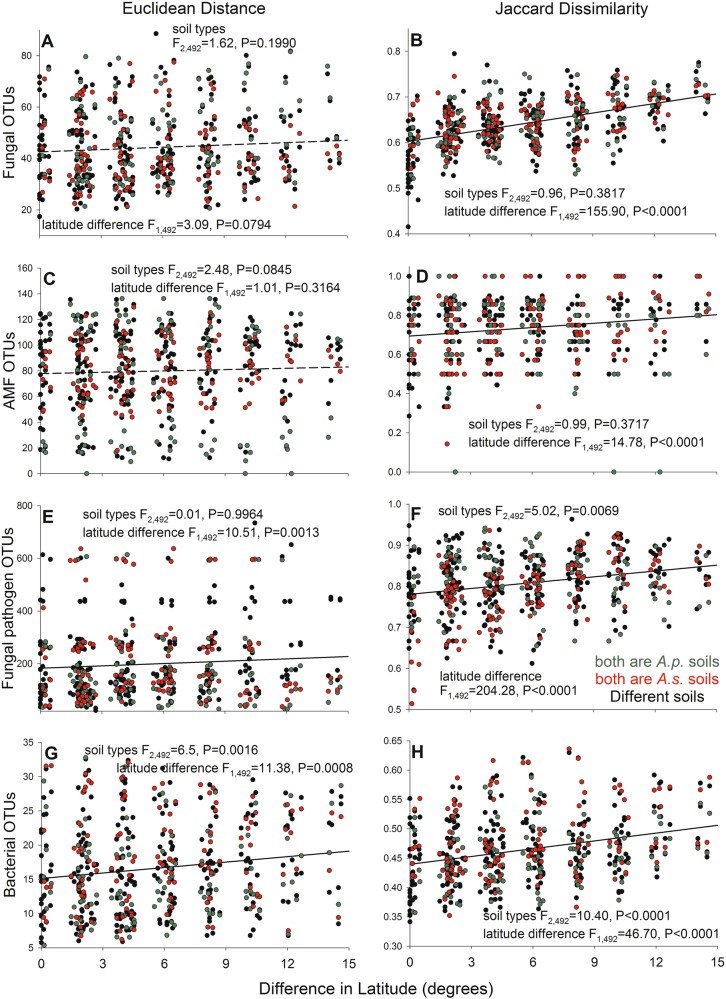
Relationship between the Euclidean Distance and Jaccard Dissimilarity of fungal OTUs (**a** and **b**), AMF OTUs (**c** and **d**), fungal pathogen OTUs (**e** and **f**), and bacterial OTUs (**g** and **h**) and the latitude difference between paired samples that both were from *A. philoxeroides* soils (green), *A. sessilis* soils (red), or from different soil types (black). The *F*-values and *P*-values are from ANCOVAs and lines indicate effects of latitude. For fungal pathogens (**f**) and bacteria (**e** and **h**), communities were most dissimilar for pairs of *A. sessilis* soils, intermediate for a pairing of different soils, and least different for pairs of *A. philoxeroides* soils in post-hoc tests

Total fungal OTU composition varied with precipitation, AMF OTU composition varied with N:P, pH, Tmax, and soil type, potential fungal pathogen OTU composition varied with TP, AP, precipitation, pH, Tmax, and Tmaxavg, and bacterial OTU composition varied with pH and N:P (Supplementary Table S3 and Supplementary Fig. S4). Soils had more dissimilar fungal (Jaccard but not Euclidean index), AMF (Jaccard only), potential fungal pathogen (both), and bacterial (both) assemblages when they were from more distant sites by latitude (Fig. 2). Soils had more dissimilar fungal pathogen assemblages when both were *A. sessilis* soils and less dissimilar fungal pathogen assemblages when both were *A. philoxeroides* soils or were different soils (Fig. 2f). Soils had the most dissimilar bacterial assemblages when both were *A. sessilis* soils, the least dissimilar bacterial assemblages when both were *A. philoxeroides* soils, and an intermediate assemblage dissimilarity when they were different soils (Figs. 2g, h).

The number of nematode knots was 8.72-fold higher on average on *A. sessilis* roots than on *A. philoxeroides* roots. The knot number decreased with increasing soil collection latitude for both plant species, but the rate of decrease was more rapid for *A. sessilis* (slopes: −1.065 for *A. sessilis* vs. −0.336 for *A. philoxeroides*), which had higher numbers of knots at lower latitudes (Table 1 and Fig. 1b). The latitude effect size was larger for *A. sessilis* (*f*^2^ = 0.672) than for *A. philoxeroides* (*f*^2^ = 0.418).

**Table 1:** Tests of the effects of soil collection latitude on plants grown in rhizosphere soil of *A. philoxeroides* or *A. sessilis* and beetles fed their leaves

**Overall**	**Plant mass**		**# of seeds**		**Sqrt (# of knots)**		**Beetle mass**	
Factor	*F*_1, 232_	*P*			*F*_1, 232_	*P*	*F*_1, 464_	*P*
Soil	0.01	0.9957			0.29	0.5939	0.34	0.5621
Species	1.79	0.1824			**43.93**	**<0.0001**	**11.39**	**0.0008**
Soil × species	0.50	0.4785			0.16	0.6907	0.02	0.8998
Latitude	**16.57**	**<0.0001**			**116.37**	**<0.0001**	**9.93**	**0.0017**
Soil × latitude	0.02	0.8939			0.54	0.4643	0.24	0.6220
Species × latitude	**8.64**	**0.0036**			**31.46**	**<0.0001**	**8.99**	**0.0029**
Soil × species × latitude	0.50	0.4781			0.25	0.6199	0.02	0.8768
*A. philoxeroides* plants								
Factor	*F*_1, 116_	*P*			*F*_1, 116_	*P*	*F*_1, 237_	*P*
Soil	0.22	0.6416			0.03	0.8557	0.10	0.7529
Latitude	0.55	0.4618			**48.48**	**<0.0001**	0.01	0.9147
Soil × latitude	0.30	0.5828			0.10	0.7511	0.06	0.8138
*A. sessilis* plants								
Factor	*F*_1, 116_	*P*	*F*_1, 116_	*P*	*F*_1, 116_	*P*	*F*_1, 227_	*P*
Soil	0.30	0.5847	0.24	0.6252	0.25	0.6165	0.26	0.6112
Latitude	**29.75**	**<0.0001**	**19.17**	**<0.0001**	**78**	**<0.0001**	**19.47**	**<0.0001**
Soil × latitude	0.20	0.6542	0.19	0.6632	0.44	0.5091	0.22	0.6422

Overall models included species of plants, soil type (rhizosphere of *A.sessilis* vs. rhizosphere of *A. philoxeroides*), latitude, and their interactions. Response variables are: mass of invasive *A. philoxeroides* or native *A. sessilis* plants, the number of seeds of the native plant, the number of nematode root knots on the invasive or native plants, and the mass of *A. hygrophila* beetles fed leaves of the invasive or native plants. Significant results are shown in bold.

**Fig. 3:**
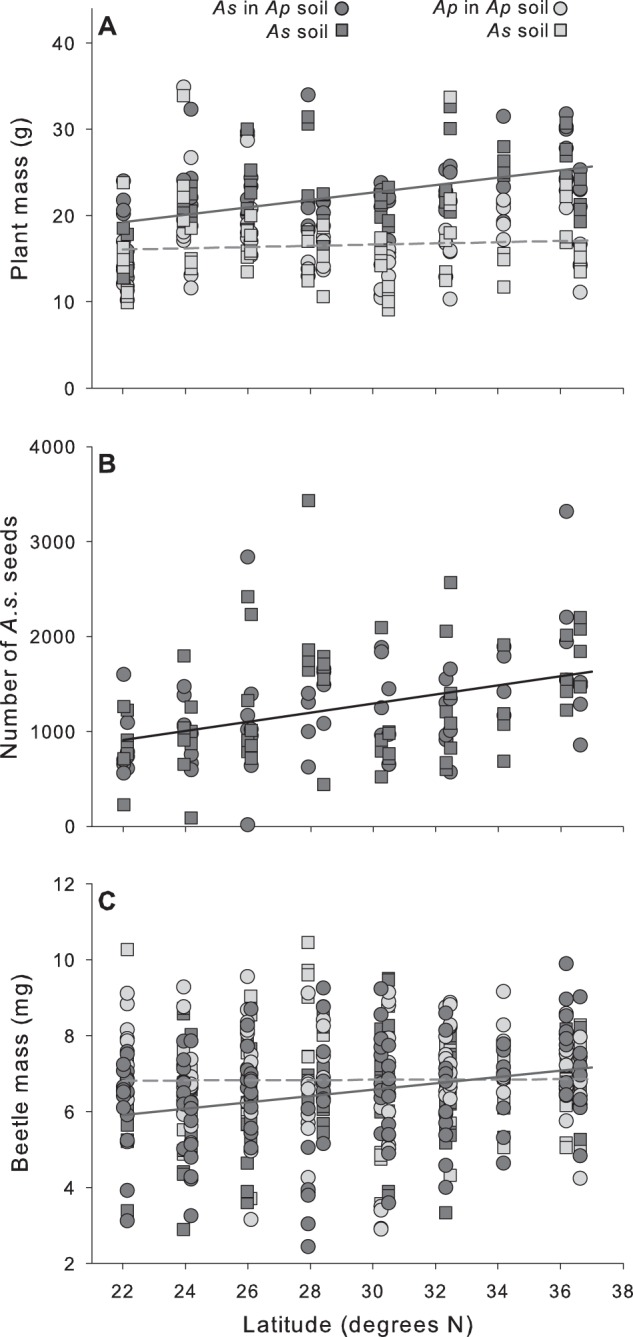
**a** Biomass of invasive *A. philoxeroides* (light gray) vs. native *A. sessilis* (dark gray) plants and **b** number of *A. sessilis* seeds for plants grown in *A. philoxeroides* (cirles) or *A. sessilis* (squares) rhizosphere soils collected along a latitudinal gradient. **c** Mass of *A. hygrophila* beetles fed leaves of the invasive or native plants grown in these soils. Solid lines indicate significant effects of latitude and dashed lines indicate non-significant relationships with latitude for each plant species

*Alternathera sessilis* mass increased by 32.8% along the latitudinal gradient of soil collection (*f*^2^ = 0.256), but *A. philoxeroides* mass did not change with soil collection latitude (Table 1 and Fig. 3a). The number of *A. sessilis* seeds increased by 20.7% along the latitudinal gradient of soil collection (Table 1 and Fig. 3b) but the magnitude of this effect was “small” (*f*^2^ = 0.165).

Beetle survival odds were independent of all predictors (Supplementary Table S1). Beetle mass was 5.2% higher on average when they ate *A. philoxeroides* than *A.sessilis* leaves; in addition, their mass increased by 20.0% along the latitudinal gradient of soil collection when they ate *A. sessilis* leaves (effect size *f*^2^ = 0.086), but soil collection latitude did not affect their mass when they ate *A. philoxeroides* leaves (Table 1 and Fig. 3c).

**Fig. 4:**
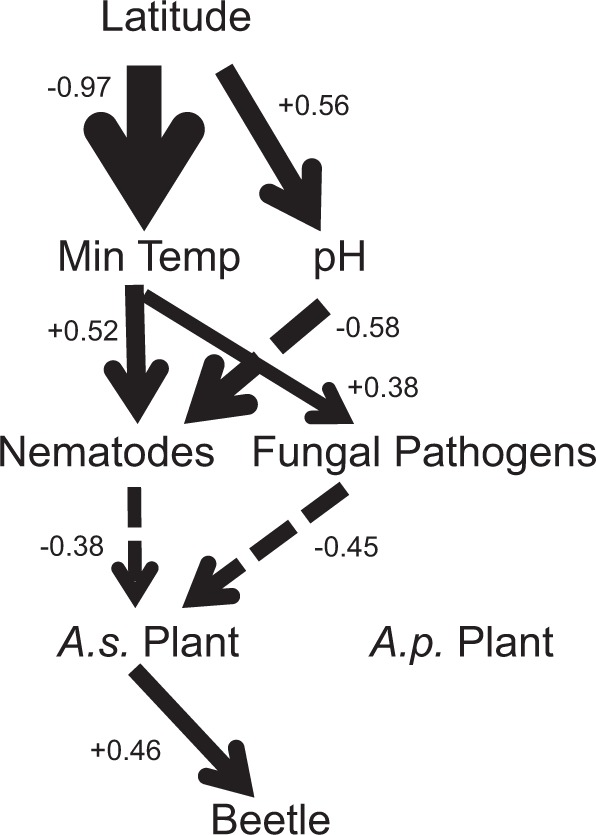
Path diagram showing: latitude effects on abiotic variables (annual minimum temperature and soil pH); abiotic variable effects on nematode knot abundance and diversity of soil fungal pathogens; nematode and pathogen effects on performance of the native and invasive plants; effects of plants on beetles fed their leaves. Only significant paths are shown. Dashed lines indicate negative effects and solid lines denote positive effects. The width of the lines indicates the magnitude of effects

Path analysis (Fig. 4) showed that the abundance of root-knot nematode and potential soil-borne fungal pathogens increased with annual minimum temperature that decreased with latitude. The increase in pH with latitude contributed to decreases in root knots. Root-knot nematodes and soil-borne fungal pathogens suppressed the performance of *A. sessilis*, which in turn suppressed beetle performance. Neither root-knot nematodes nor soil fungal pathogens had an impact on *A. philoxeroides*.

## Discussion

Release from soil-borne pathogens and herbivores or enhanced positive soil interactions via mutualists are considered important factors contributing to plant invasions [21]. Testing how latitudinal changes in soil biota communities affect native and invasive plants may help to understand latitudinal variation in biotic interactions and predict the strength of enemy release or biotic resistance of invasive plant species. Here, we show that the native plant performance increased with soil collection latitude, consistent with the Latitudinal Biotic Interaction Hypothesis, likely due to the decreasing abundance and diversity of soil-borne enemies. However, as predicted, there was no latitudinal pattern for the invasive plant mass. In addition, we found soil biotic effects cascaded to above-ground beetle performance only when the beetles were fed foliage of the native plant rather than the invasive plant. These novel results highlight the importance of linking above- and below-ground trophic interactions when exploring the role of soil biota in shaping plant performance along latitudinal gradients. They also add to an emerging body of literature [15–19] that emphasizes the need to study species invasions within a biogeographic context.

Plant invasions may alter soil communities, which in turn may reinforce their invasions or favor subsequent invasions by other non-native species [21, 46, 47]. In our study, we did not find clear differences between the native and invasive rhizosphere communities for most groups. However, the greater dissimilarity of bacterial and fungal pathogen assemblages for pairs of native plant soils and greater similarity for pairs of invasive plant soils suggest that invasive rhizosphere communities are composed of a subset of general, broadly distributed bacterial and fungal pathogens, whereas native assemblages contain rarer and more narrowly distributed taxa. Furthermore, AMF composition differed between the native and invasive plant rhizospheres in this study, which is consistent with other studies [48, 49].

Native plant growth, reflecting the net effects of antagonistic and mutualist interactions, increased with increasing latitude of soil collection. This was likely due to decreasing negative impacts of soil-borne enemies with soil collection latitude based on the following evidence. First, our path analysis found a negative association between both soil fungal pathogen diversity and root-knot nematode abundance and native plant performance. Second, soil-borne enemy abundance (root-knot nematodes) and diversity (fungal pathogens) decreased with latitude, which is consistent with the Latitudinal Biotic Interaction Hypothesis [5, 9], whereas no latitudinal pattern was observed for AMF, bacteria, or fungi in general. However, changes in the taxonomic composition of bacteria, pathogenic fungi, and AMF communities with latitude indicate that species differences in the strengths of negative and/or positive interactions might also contribute to the pattern we and others [28, 29] have found, i.e., negative effects of the soil biota diminishing at higher latitudes.

In contrast to the Latitudinal Biotic Interaction Hypothesis, we found no latitudinal pattern in plant–soil interactions for the invasive plant. Our path analysis only detected a negative association between soil enemy abundance and plant performance for the native plant, not for the invasive plant, likely reflecting the invasive plant’s more effective defense against fungal pathogens or nematode attacks [35]. In addition, relative to the long evolutionary history of the native plant with the soil biota, the invasive plant had a short soil association history (less than 100 years), likely lacking sufficient time to evolve the observed gradient. Furthermore, unlike the native plant which can reproduce by seeds that may generate genetic variation during the evolutionary process, the invasive plant only reproduces by vegetative propagation, likely decreasing the latitudinal variation in its interactions with soil biota. Contrasting latitudinal patterns of soil biotic effects on the native vs. invasive plants may create spatial variation in the strengths of enemy release and biotic resistance that the invasive plant will experience during range expansion. At low latitudes, a higher defense might enable the invasive plant to escape more from soil-borne enemies than the native plant, whereas at high latitudes the invasive plant might experience increasing resistance from native competitors due to the decreased impact of soil-borne enemies.

The impacts of soil biota on plants could cascade to above-ground herbivores through food webs, a phenomenon that has been extensively studied at local scales but has not been tested across biogeographic gradients. Here, we provided the first study of this kind, showing the larval mass of *A. hygrophila* fed native plant leaves increased as a function of soil collection latitude, but the larval mass of beetles fed invasive plant leaves did not. Path analysis indicated soil fungal pathogens and nematodes had a negative impact on *A. hygrophila* via the native plant, but found no impact of soil biota on *A. hygrophila* via the invasive plant, which is consistent with our previous finding [50]. Soil biota could affect above-ground herbivores by altering plant defense and/or nutritional content [51]. In a previous study, we found differing impacts of soil nematode *M. incognita* infection on the native beetle *Cassida piperata* via *A. philoxeroides* and *A. sessilis*, and detected a minor impact of soil nematodes on the foliar C:N of both plant species [52]. Whether the observed effects of the soil biota on *A. hygrophila* were due to nutritional or defensive changes induced by soil-borne enemies and how the two host plants differ in mediating these effects need to be further studied with experiments involving sterilized soils.

Our investigation of soil–plant–herbivore interactions along a latitudinal gradient is limited by the single native-invasive plant pair and future tests will require more native and invasive plant species. Despite this limitation, the differing latitudinal patterns in plant–soil interactions found in this study might also be valid for other species and types of biotic interactions (e.g., plant–insect/pathogen interactions, plant–pollinator interactions). Increasing evidence shows that the abundance or diversity of soil-borne enemies and above-ground herbivores or predators decreases with latitude [5, 26]. As a result, the strength of biotic interactions, including plant–soil interactions, plant–herbivory interactions, and prey–predator interactions, decreases with latitude for many native species [7, 8, 28, 29]. Moreover, a greater defense of invasive plants than native plants against soil-borne enemies has been documented in other systems [22], and biotic interactions involving invasive plants are characterized by evolutionary novelty [16]; thus, an absence of latitudinal patterns in these systems is likely but needs further study.

The differing latitudinal patterns of soil–plant–herbivore interactions for native and invasive plants we observed have important implications for understanding plant range expansions or invasions across latitudes under current and future climates if these patterns apply to other plant species and types of biotic interactions. Release from soil-borne and above-ground enemies has been proposed as an important factor underlying plant invasions [12] and species range expansions to high latitudes [53]. Given a lower dispersal rate of soil biota than plants [54], our results indicate plants will likely experience decreasing soil-borne enemies during poleward range expansions. According to our findings, soil-borne enemy release at higher latitudes is expected to facilitate range expansion of susceptible species that experience more negative impacts at low latitudes, but this facilitation via enemy release might be weak or nonexistent for plant species that experience weaker negative or perhaps even positive impacts at low latitudes. This might also be the case for above-ground herbivory when herbivores disperse slowly. In addition, our use of a single source population for native plants might have overestimated ecological effects of variation in enemy abundance, but may have underestimated effects of variation in enemy abundance through evolutionary responses (including coevolution of plants and specialist herbivores) if plant defense and growth allocation patterns reflect latitudinal differences in enemy attack [16, 55]. This opens the possibility that more poorly defended high latitude populations from the native range could establish in and invade the lower latitudes of their introduced ranges.

Furthermore, soil biotic effects on plant invasions or range expansions, indirectly affected by above-ground herbivory, have been largely overlooked. Currently, *A. hygrophila* occurs in a region below 30.8 ^o^N in continental China, but climate warming could trigger an expansion of the range to higher latitudes [37] and increase its non-target attacks on *A. sessilis* [33]. This study indicates that, during *A. hygrophila*’s northward range expansion, its performance may be improved when consuming *A. sessilis* due to changes in the soil biota. Although highly significant, the size of the effect of soil collection latitude on the mass of beetles fed native plant foliage was low. More evidence is needed to understand the importance of this pattern. In addition, it remains an open question whether indirect soil biotic effects on herbivores will affect competition between native and invasive plants, which depends on the relative strength of direct and indirect effects. Thus, to explore the role of soil biota in plant invasions and range expansion, it is critical to fully assess direct and indirect (via above-ground herbivores) effects on plants within a biogeographic context.

Currently, most theories and studies on biological invasions assume that biotic interactions are uniform across the geographic distributions of species in their invaded ranges, with few exceptions [16–18]. However, we found that latitudinal variation in the interactions among the soil microbes, soil herbivores, plants, and foliar herbivores differed for native and invasive plants, which potentially creates spatial variation in enemy release or biotic resistance and in turn potentially influences invasion dynamics. Moreover, the latitudinal decrease in the diversity of soil-borne pathogens and the abundance of root-knot nematodes, which is partially driven by minimum temperature, suggests that current and future climate change could affect plant–soil biota interactions over latitudes. Thus, considering the variation in these complex interactions across latitudes will help to better understand and predict invasion patterns and their ecological impacts at large scales. Overall, our results highlight the roles of plant defense and latitudinal variation in microbial communities in shaping biotic interactions and the importance of linking above- and below-ground multitrophic interactions while testing the Latitudinal Biotic Interaction Hypothesis.

## Electronic supplementary material

Supplemental information
