# Supplementary material for: Latitudinal variation in soil biota: testing the biotic interaction hypothesis with an invasive plant and a native congener
Source: ISME J. 2018 Jul 16;12(12):2811–22. doi: 10.1038/s41396-018-0219-5 (PMC6246596; doi:10.1038/s41396-018-0219-5)
Supplement: Supplementary file 1 — Supplemental information [file 41396_2018_219_MOESM1_ESM.doc]

**Supplementary Information**

**Table S1** Sites used in the analysis of soil biota (and physical attributes), greenhouse experiment, and bioassay experiment. For the XC site, the amount of soil after screening was not sufficient for the greenhouse experiment and so it was excluded. Numbers indicate beetles surviving in the bioassay experiment out of 10 initial larvae. Some sets of leaves were lost in a refrigerator malfunction (indicated by n/a). The odds of beetle survival did not differ between *A. philoxeroides* vs. *A. sessilis* soils (*F*_1, 47_=0.08, *P*=0.7822), or *A. philoxeroides* vs. *A. sessilis* plants (*F*_1, 47_=0.04, *P*=0.8429), or among combinations of soil and plant types (*F*_1, 47_=0.01, *P*=0.9268). Survival did not depend on latitude as a main effect (*F*_1, 47_=0.36, *P*=0.5505) or in interaction with soil type (*F*_1, 47_=0.55, *P*=0.4603), plant type (*F*_1, 47_=2.12, *P*=0.1520), or their combination (*F*_1, 47_=2.43, *P*=0.1256).

|  |  |  |  |  |  | |  | |
| --- | --- | --- | --- | --- | --- | --- | --- | --- |
|  |  |  |  |  | Beetles in *Ap* soil | | Beetles in *As* soil | |
| site | Latitude | Soil | Greenhouse | Bioassay | *Ap* plant | *As* plant | *Ap* plant | *As* plant |
| YC | 22.14 | X | X | X | 9 | 10 | 9 | 8 |
| QY | 23.95 | X | X | X | 9 | 8 | 8 | 9 |
| GX | 24.18 | X | X | X | 9 | 9 | 8 | 7 |
| CZ | 25.98 | X | X | X | 10 | 5 | 10 | 8 |
| YX | 26.10 | X | X | X | 7 | 10 | 8 | 9 |
| YJ | 26.10 | X | X | X | n/a | 7 | n/a | n/a |
| HN | 27.93 | X | X | X | 9 | 6 | 9 | 9 |
| CS | 28.41 | X | X | X | 10 | 9 | 9 | 9 |
| JX | 30.26 | X | X | X | 6 | 9 | 8 | 10 |
| WH | 30.50 | X | X | X | 10 | 10 | 6 | 7 |
| HF | 32.33 | X | X | X | 9 | 10 | 9 | 8 |
| XY | 32.47 | X | X | X | 10 | n/a | 9 | 10 |
| XC | 33.93 | X |  |  |  |  |  |  |
| XZ | 34.18 | X | X | X | 8 | 8 | 9 | n/a |
| JN | 36.18 | X | X | X | 8 | 10 | 10 | 8 |
| TY | 36.63 | X | X | X | 6 | 9 | 9 | 9 |
|  |  |  |  |  |  |  |  |  |

**Table S2** The dependence of soil chemistry variables and the number of soil biota OTUs on soil type [rhizosphere of *A. sessilis* (*As* soil) *or A. philoxeroides* (*Ap* soil)], latitude (normalized so zero is the middle of the latitude gradient), and their interaction. F- values, P-values, and local effect sizes (Cohen’s *f*^2^). Terms with significant P-values (<0.05) and non-trivial effect sizes (*f*^2^>0.1) are shown in bold.

|  |  |  |  |  |  |  |  |  |  |
| --- | --- | --- | --- | --- | --- | --- | --- | --- | --- |
|  | Soil type | | | Latitude | | | Soil type × latitude | | |
| Variable | *F*_1, 29_ | *P* | *f*^2^ | *F*_1, 29_ | *P* | *f*^2^ | *F*_1, 29_ | *P* | *f*^2^ |
| TC | 0.96 | 0.3361 | 0.034 | 1.34 | 0.2565 | 0.048 | 1.02 | 0.3207 | 0.037 |
| TN | 1.10 | 0.3023 | 0.039 | 1.55 | 0.2237 | 0.055 | 1.11 | 0.3009 | 0.040 |
| TP | 0.44 | 0.5141 | 0.016 | 0.76 | 0.3907 | 0.027 | 0.52 | 0.4785 | 0.018 |
| C:N | 0.20 | 0.6582 | 0.007 | 1.10 | 0.3041 | 0.039 | 0.26 | 0.6158 | 0.009 |
| C:P | 0.48 | 0.4952 | 0.017 | 0.09 | 0.7618 | 0.003 | 0.48 | 0.4933 | 0.017 |
| N:P | 0.20 | 0.6568 | 0.007 | 0.08 | 0.7728 | 0.003 | 0.16 | 0.6949 | 0.006 |
| AN | 0.08 | 0.7785 | 0.003 | 0.29 | 0.5941 | 0.010 | 0.03 | 0.8562 | 0.001 |
| AP | 0.01 | 0.9205 | 0.001 | 0.94 | 0.3418 | 0.033 | 0.03 | 0.8603 | 0.001 |
| OC | 0.65 | 0.4252 | 0.023 | 0.01 | 0.9635 | 0.001 | 0.69 | 0.4127 | 0.025 |
| pH | 0.82 | 0.3737 | 0.029 | **12.96** | **0.0012** | **0.463** | 0.71 | 0.4050 | 0.026 |
| # Fungus OTUs | 0.04 | 0.8369 | 0.002 | 0.76 | 0.3912 | 0.027 | 0.07 | 0.7923 | 0.003 |
| Pathogens | 0.14 | 0.7089 | 0.005 | **7.33** | **0.0114** | **0.262** | 0.07 | 0.7872 | 0.003 |
| AMF | 0.51 | 0.4818 | 0.018 | 0.02 | 0.9028 | 0.001 | 0.99 | 0.3277 | 0.035 |
| # Bacteria OTUs | 0.02 | 0.9032 | 0.001 | **6.55** | **0.0162** | **0.234** | 0.13 | 0.7260 | 0.004 |
|  |  |  |  |  |  |  |  |  |  |

**Table S3** Factors that significantly explained the variation in the first three PCA axes for different soil microbial groups. Starting models included soil chemistry variables (TC, TN, TP, C:N, C:P, N:P, AN, AP, OC, pH), climate variables (Tmin, Tmax, Tminavg, Tmaxavg, Tmedian, precipitation), and plant species occurring where soil was collected (“soil”). Stepwise, forward, and backward model selection each selected the same final model for each PCA axis. P-values for significant terms are shown in brackets and F-values are shown in parentheses. Signs indicate relative directions of effects for axes with more than one significant predictor.

|  |  |  |  |
| --- | --- | --- | --- |
|  | Axis 1 | Axis 2 | Axis 3 |
| Fungus OTUs | Precip [0.0164] | Precip [0.0248] | none |
|  | (*F*_1, 30_=6.46) | (*F*_1, 30_=5.58) |  |
|  |  |  |  |
| AMF OTUs | -pH [0.0054] | +Soil [0.0142] | pH [0.0295] |
|  | (*F*_1, 29_=9.04) | (*F*_1, 29_=6.81) | (*F*_1, 30_=5.22) |
|  |  |  |  |
|  | +N:P [0.0237] | +Tmax [0.0317] |  |
|  | (*F*_1, 29_=5.70) | (*F*_1, 29_=5.09) |  |
|  |  |  |  |
| Fungal pathogen OTUs | Precip [0.0003] | +AP [<0.0001] | +Tmaxavg [0.0002] |
|  | (*F*_1, 30_=16.71) | (*F*_1, 29_=24.30) | (*F*_1, 28_=18.28) |
|  |  |  |  |
|  |  | -TP [0.0251] | -Tmax [0.0019] |
|  |  | (*F*_1, 29_=5.59) | (*F*_1, 28_=11.75) |
|  |  |  |  |
|  |  |  | +pH [0.0113] |
|  |  |  | (*F*_1, 28_=7.56) |
|  |  |  |  |
| Bacteria OTUs | none | pH [0.0003] | N:P [0.0274] |
|  |  | (*F*_1, 30_=16.40) | (*F*_1, 30_=5.38) |
|  |  |  |  |
|  | | | |

**
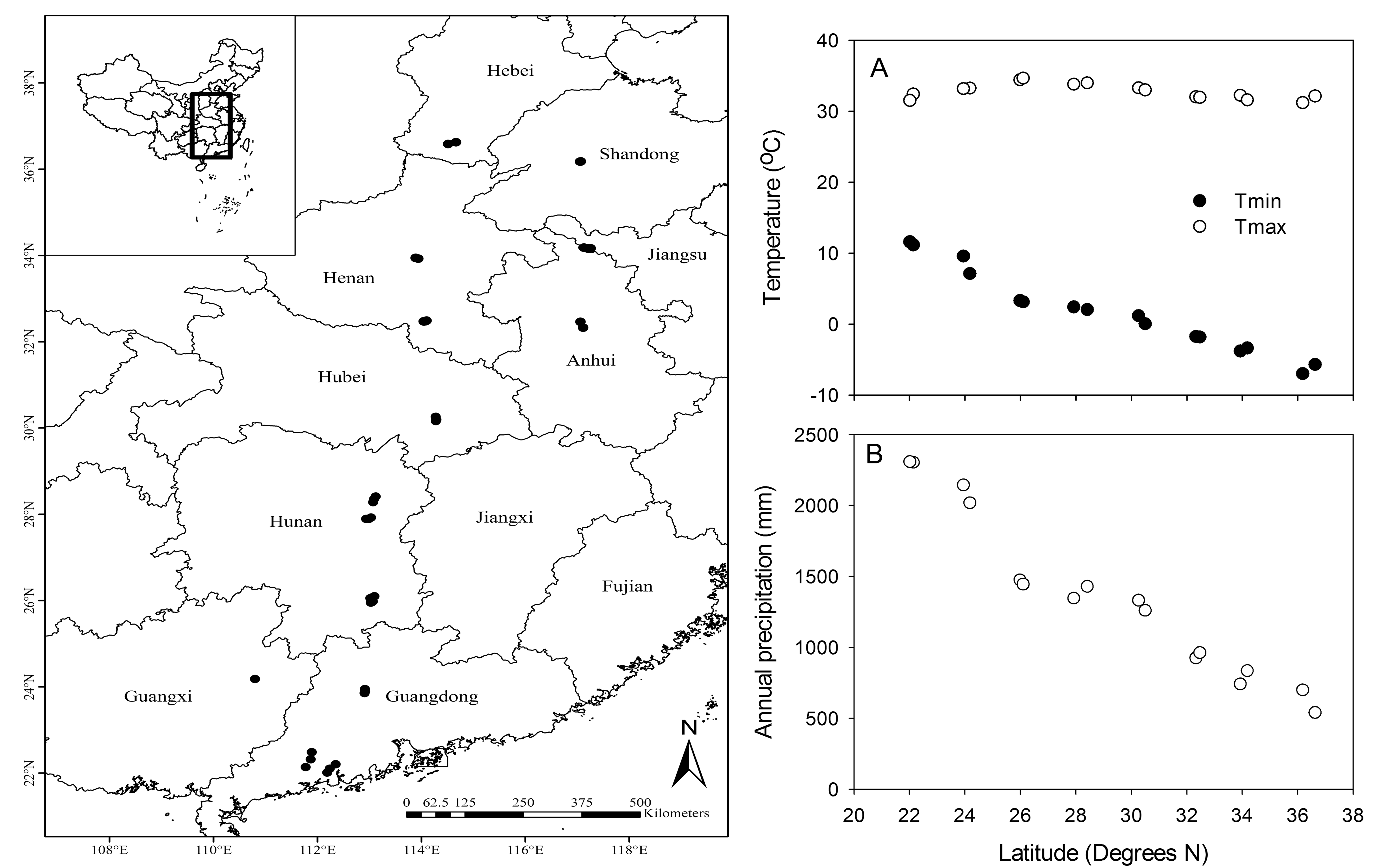
**

**Fig. S1** The sites where soil samples were collected in this study (left), and the annual minimum (Tmin) and maximum (Tmax) temperatures (A, right), and annual precipitation (B, right) of these sites along the latitudinal gradient.

**Fig.S2** pH values of the soil samples collected from invasive *A. philoxeroides* (green) and native *A. sessilis* (red) rhizospheres along the latitudinal gradient for this study.

**Fig S3** (A) Total nitrogen, (B) water soluble nitrogen, (C) total carbon, (D) organic carbon, (E) total phosphorus, and (F) available phosphorus contents of soil samples collected from invasive *A. philoxeroides* (green) and native *A. sessilis* (red) rhizospheres along the latitudinal gradient for this study.

**Fig. S4** Principal component analysis results for soil samples collected from invasive *A. philoxeroides* (green) and native *A. sessilis* (red) rhizospheres along the latitudinal gradient (numbers are degrees north) for this study: (A) environmental variables by soil samples, (B) environmental variables by sites, (C) fungal OTUs by soil samples, (D) AMF OTUs by soil samples, (E) fungal pathogen OTUs by soil samples, and (F) bacterial OTUs by soil samples.
